# Identification of Novel Subcellular Localization and Trafficking of HIV-1 Nef Variants from Reference Strains G (F1.93.HH8793) and H (BE.93.VI997)

**DOI:** 10.3390/v10090493

**Published:** 2018-09-13

**Authors:** Logan R. Van Nynatten, Aaron L. Johnson, Brennan S. Dirk, Emily N. Pawlak, Rajesh Abraham Jacob, S. M. Mansour Haeryfar, Jimmy D. Dikeakos

**Affiliations:** Department of Microbiology and Immunology, The University of Western Ontario, Schulich School of Medicine and Dentistry, London, ON N6A 5C1, Canada; lvannyna@uwo.ca (L.R.V.N.); ajohn228@uwo.ca (A.L.J.); bdirk@uwo.ca (B.S.D.); epawlak@uwo.ca (E.N.P.); rjacob24@uwo.ca (R.A.J.); mansour.haeryfar@schulich.uwo.ca (S.M.M.H.)

**Keywords:** human immunodeficiency virus, Nef, diversity, subtypes, microscopy

## Abstract

The human immunodeficiency virus type 1 (HIV-1) accessory protein Nef, plays an essential role in disease progression and pathogenesis via hijacking the host cellular membrane-trafficking machinery. Interestingly, HIV-1 group-M subtypes display differences in the rate of disease progression. However, few reports investigated how the cellular behaviors and activities of Nef isolates from reference strains may differ between HIV-1 group-M subtypes. Here, we characterize how differing cellular distributions of Nef proteins across group-M subtypes may impact protein function using immunofluorescence microscopy and flow cytometric analysis. We demonstrate that Nef variants isolated from HIV-1 group-M subtypes display differences in expression, with low expressing Nef proteins from reference strains of subtypes G (F1.93.HH8793) and H (BE.93.VI997) also displaying decreased functionality. Additionally, we demonstrate variations in the subcellular distribution and localization of these Nef proteins. Nef from subtype G (F1.93.HH8793) and H (BE.93.VI997) reference strains also failed to colocalize with the trans-Golgi network, and were not differentially localized to cellular markers of multivesicular bodies or lysosomes. Strikingly, our results demonstrate that HIV-1 Nef proteins from reference strains G (F1.93.HH8793) and H (BE.93.VI997) highly colocalize with labeled mitochondrial compartments.

## 1. Introduction

The human immunodeficiency virus type 1 (HIV-1) demonstrates vast viral genetic diversity due to its rapid viral mutation rate. Accordingly, this HIV-1 diversity can be classified into different phylogenetic groups termed M, N, O, or P, which are defined according to the degree of sequence homology [1]. Group M accounts for over 90% of global infections, and can be further classified into nine subtypes, A through K (no I or E) [1,2]. Subtype A predominates in West Africa and Eastern Europe, while subtype B is prevalent in the Americas and Western Europe [3,4,5]. In sub-Saharan Africa, subtype C accounts for most infections, while in Central Africa—which is the origin of the HIV epidemic—all subtypes are prevalent [3,4,5]. Subtype G is more prominent in West Africa, while subtypes H, J, and K are commonly found in Central, Southern, and West Africa [2].

With the shift toward global control of the epidemic, much interest is placed on understanding the impact of HIV-1 genetic diversity on clinical outcomes. Indeed, disease progression and patient outcomes may vary depending on the infecting HIV-1 subtype [6,7,8,9]. Notably, subtype C displays a slower disease progression, while subtype D was reported to display accelerated disease progression upon infection [6,10,11]. However, there is little evidence suggesting why these differences in disease progression occur. Accordingly, with this high viral genetic diversity comes diversity in the different HIV-1 genes, including the gene encoding the HIV-1 accessory protein Nef. It is reported that the HIV-1 accessory protein, Nef, plays a critical role in disease progression and pathogenesis. Based on the essential role Nef plays in pathogenesis [12,13,14,15], it is valuable to begin to comprehend the function and cellular behaviors of Nef variants from less common subtypes, and how they compare to more predominant subtypes.

Specifically, the HIV-1 accessory protein, Nef, despite lacking any known enzymatic activity, is instrumental in facilitating HIV-1 infection through manipulating the host cellular membrane-trafficking machinery, as well as signal transduction cascades, to promote viral pathogenesis and host immune evasion [16,17,18]. This hijacking of the membrane-trafficking machinery largely involves downregulating immune cell surface receptors—namely, cluster of differentiation 4 (CD4) and major histocompatibility complex class I (MHC-I)—to prevent killing of HIV-1-infected cells [17,19]. This Nef-mediated cellular hijacking is critically dependent on the ability of Nef to traffic and localize to various subcellular compartments.

Upon expression in host cells, Nef binds the membrane-trafficking regulator proteins, phosphofurin acidic cluster proteins 1 and 2 (PACS1/2), to promote endocytosis and downregulation of major histocompatibility complex class I (MHC-I) molecules from the cell surface [20,21]. This activity is dependent on the PACS-2-mediated trafficking and localization of Nef to the trans-Golgi network (TGN), where Nef directly binds and activates Src-family kinase proteins, thus activating a signal transduction pathway that promotes MHC-I endocytosis [20,21]. Subsequently, internalized MHC-I [22] is trafficked and sequestered in the TGN in a PACS-1-dependent process [23]. Overall, the Nef-dependent downregulation of MHC-I results in viral immune evasion, preventing elimination of HIV-1-infected cells by cytotoxic T lymphocytes (CTLs).

Alternatively, Nef-mediated downregulation of the main receptor for HIV-1 entry, CD4, from the cell surface prevents cellular re-infection and inhibits antibody-dependent cell cytotoxicity (ADCC) [22,24,25,26]. Specifically, this downregulation is facilitated by the Nef/adaptor protein 2 (AP-2)/CD4 complex, to which Nef localizes on the cell surface [24,27,28]. Subsequently, localization of Nef to coat protein (COP-I)-coated vesicles allows for Nef to mediate trafficking of CD4 from early to late endosomes [29]. Ultimately, Nef’s hijacking of specific cellular compartments mediates CD4 trafficking to lysosomes for degradation [29,30,31].

Overall, the Nef-mediated subcellular trafficking events that facilitate the downregulation of CD4 and MHC-I demonstrate the importance of proper localization of Nef to cellular compartments and trafficking machinery. Indeed, Nef’s ability to bind to and localize to specific cellular compartments is crucial for its ability to hijack the trafficking machinery. Combining this with the extensive HIV-1 viral genetic diversity, we sought to investigate how the genetic diversity of HIV-1 impacts Nef function and subcellular localization across HIV-1 group-M subtypes. Specifically, we characterized the novel subcellular localization of HIV-1 Nef proteins from reference strains G (F1.93.HH8793) and H (BE.93.VI997), and evaluated the cellular expression and efficiency of these strains in performing essential Nef functions.

## 2. Materials and Methods

### 2.1. Cell Culture

CD4 HeLa (American Type Culture Collection (ATCC), Manassas, VA, USA) and HEK 293T cells (Life Technologies, Carlsbad, CA, USA) were grown in Dulbecco’s modified Eagle medium (DMEM) containing 10% fetal bovine serum (FBS; Wisent, QC, Canada) and 1% penicillin and streptomycin (Hyclone, Logan, UT, USA). Jurkat E6.1 T cells (Catalog number 177; National Institutes of Health, acquired immune deficiency syndrome (AIDS) Research and Reference Reagent Program) were cultured in Roswell Park Memorial Institute (RPMI) medium 1640 containing 10% FBS, 1% penicillin and streptomycin, 1% l-glutamine, 1% non-essential amino acids, and 1% sodium pyruvate. All cell types were grown in 37 °C and 5% CO_2_, and sub-cultured based on ATCC recommendations.

### 2.2. Pseudovirus Production and Viral Transduction

Pseudovirions were produced in HEK 293T cells via a triple transfection with pNL4.3 Δgag/pol enhanced GFP (eGFP), pdR8.2 (Addgene; catalog number 12263), and pMD2.G (Addgene; catalog number 12259) plasmids, using PolyJet (FroggaBio, Toronto, ON, Canada), as previously reported [32]. Different eGFP-tagged or untagged Nef proteins were subcloned into the pNL4.3 Δgag/pol eGFP proviral backbone as previously described [32,33] using an AgeI/NotI restriction digest. The pseudovirus was collected 48 h post transfection, with virus-containing media centrifuged at 3000× *g* for 5 min and subsequently filtered with a 0.2-μm filter. The supernatant was supplemented with an additional 10% FBS before storage at −80 °C. To transduce Jurkat E6.1 T cells with pseudovirus (only differing in the *nef* gene from reference strains of group-M subtypes), 8 × 10^5^ Jurkat E6.1 T cells were pelleted for 10 min at 1200 rpm. The supernatant was subsequently removed, and the cell pellet resuspended in 800 µL of pseudovirus, along with 200 µL of FBS and 8 µL of Polybrene (8 µg/mL; Sigma Aldrich Co, St Louis, MO, USA). Cells were then aliquoted into 12-well plates and incubated at 37 °C. Infections were allowed to proceed for 48 h prior to analysis via immunoblotting or flow cytometry. To transduce HeLa cells prior to mitochondrial staining, a mixture containing 500 μL of the respective virus, 200 μL of FBS, 8 μL of polybrene, and DMEM with 10% FBS was added to cells. After 24 h, cells were stained with MitoTracker^®^ Deep Red FM (Thermo Fisher Scientific; Pittsburgh, PA, USA, see below) and fixed with 4% paraformaldehyde (PFA) for imaging.

### 2.3. Immunoblotting

To immunoblot for Nef variants from various HIV-1 group-M subtypes, transduced Jurkat E6.1 T cells were infected with pseudovirus for 48 h, and transfected HeLa cells were washed once with phosphate-buffered saline (PBS) and incubated in lysis buffer (0.5 M 4-(2-hydroxyethyl)-1-piperazineethanesulfonic acid (HEPES), 1.25 M NaCl, 1 M MgCl2, 0.25 M ethylenediaminetetraacetic acid (EDTA), 0.1% Triton X-100, and 1× Complete Protease Inhibitor Tablets (Roche, Indianapolis, IN, USA)). Specifically, cells were rocked for 20 min at 4 °C before centrifugation at 20,000× *g* for 20 min, and the supernatant collected. Lysates were then boiled at 98 °C in 5× SDS-PAGE sample buffer (0.312 M Tris pH 6.8, 25% 2-mercaptoethanol, 50% glycerol, and 10% SDS) and proteins were separated on a 12% SDS-PAGE gel, after which they were transferred to nitrocellulose membranes. Membranes were blocked in 5% non-fat skim milk (Bioshop, Burlington, ON, Canada) in Tris-buffered saline/Tween (TBST) containing 0.1% Triton X-100 for 1 h, then incubated overnight at 4 °C with various antibodies: rabbit anti-Nef polyclonal antibody (1:2000; catalog number 2949, NIH AIDS Research and Reference Reagent Program, Gaithersburg, MD, USA), rabbit anti-GFP polyclonal antibody (1:2000; Clontech; Mountain View, CA, USA), and mouse anti-actin (1:2000; Thermo Fisher Scientific). Membranes were then washed and incubated for two hours with species-specific horse radish peroxidase (HRP)-conjugated antibodies (1:4000; Thermo Fisher Scientific). All blots were developed and quantified using enhanced chemiluminescence (ECL) peroxidase substrate (Millipore Inc., Billerica, MA, USA) and a C-DiGit chemiluminescence Western blot scanner (LI-COR Biosciences, Lincoln, NE, USA).

### 2.4. Flow Cytometry

Cell surface MHC-I and CD4 expression levels were quantified in transduced Jurkat E6.1 cells or transfected HeLa cells, respectively, using flow cytometry. Cells were infected or transfected, and 48 h later, fixed in 2% paraformaldehyde. Cells were stained for MHC-I with W6/32 (anti-MHC-I, panselective, provided by D. Johnson, Oregon Health and Sciences University) or for CD4 with an allophycocyanin (APC)-conjugated anti-CD4 monoclonal antibody (Clone OKT4, Biolegend, San Diego, CA, USA). Cell surface MHC-I or CD4 levels were then quantified using flow cytometry (BD FACSCanto II; BD Biosciences, Franklin Lakes, NJ, USA). Data were analyzed using FlowJo software (version 9.6.4; TreeStar, Ashland, OR, USA). Infected cells were gated on by selecting for eGFP positive cells. MHC-I downregulation efficiency (%NL4.3 = (MFI_exp_ − MFI_dNef_/MFI_NL4._ − MFI_dNef_) × 100%), and CD4 downregulation efficiency (%NL4.3 = (MFI_exp_ − MFI_egfp_/MFI_NL4.3_ − MFI_dNef_) × 100%) were calculated as previously described [33]. Downregulation efficiency is relative to NL4.3.

To quantify Nef expression levels via flow cytometry, Jurkat E6.1 cells were infected with pNL4.3 Δgag/pol Nef–eGFP lentiviral vectors and fixed 48 h later in 2% paraformaldehyde. Fixed cells were then analyzed for GFP fluorescence using flow cytometry (BD FACSCanto II). Nef-expressing cells were gated by GFP fluorescence. Subsequently, Nef expression was quantified using mean fluorescence intensity (MFI) of GFP-positive cells. Statistical analysis comparing GFP MFI was completed with GraphPad Prism using a one-way Anova with a Dunnett’s multiple comparisons test.

### 2.5. Transfections and Microscopy

Plasmids utilized for transfections and subsequent microscopy include pN1 (Clontech) Nef–eGFP plasmids encoding Nef variants from various HIV-1 group-M subtypes. These plasmids were produced from expression vectors encoding Nef proteins from isolates of HIV-1 group-M reference strains A1.SE94, A2.97CDKTB4B, B.JRFL, C.BR92025, F1.BE93VI850, F2.CM95MP257, G.FI93HH8793, H.BE.93.VI997, J.SE93SE7887, or K.CD97EQTB11C, and were provided by T. Smithgall, University of Pittsburgh. Additional G and H reference strains G (BE.1996.D) and H (CF.1990.05), in addition to a consensus D protein, were selected from the curated subtype reference alignments from the NIH Los Alamos HIV database (https://www.hiv.lanl.gov/cgi-bin/NEWALIGN/align.cgi) and ordered from GeneArt (Thermo Fisher Scientific). A pN1 red fluorescent protein (RFP)–KDEL expression vector, encoding RFP with a prolactin signal peptide sequence and a KDEL endoplasmic reticulum retention signal, and intermembrane space protein (IMS)–GFP [34] were provided courtesy of D. Heinrichs, University of Western Ontario. This pN1 RFP–KDEL vector was used to subclone and create the pN1 GFP–KDEL vector. The pN1 PACS1–eGFP was produced from complementary DNA (cDNA) obtained from G. Thomas (University of Pittsburgh), subcloned into peGFP-N1 using KpnI and XhoI restriction sites.

HeLa cells were seeded onto coverslips at 5 × 10^5^ cells/coverslip prior to transfection. The respective plasmids were transfected into HeLa cells at a concentration of 400 ng/µL using PolyJet (FroggaBio). Twelve or 24 h post transfection, cells were washed three times with PBS before fixation in 4% paraformaldehyde for 20 min at 22 °C. Cells were blocked in 5% bovine serum albumin in PBS containing 0.01% TritonX-100 for 1 h prior to immunostaining. Cells were washed again with PBS three times before immunostaining. Primary antibodies were diluted in 5% bovine serum albumin in PBS containing 0.01% TritonX-100, and cells were stained with respective antibodies for 2 h. Primary antibodies included mouse-anti-TGN46 (1:100; Sigma Aldrich, Clone TGN46-8), mouse-anti-CD63 (1:200, Developmental Studies Hybridoma Bank, Clone H5C6), mouse-anti-lysosome-associated membrane protein 1 (LAMP1; 1:200, Developmental Studies Hybridoma Bank, Clone H4A3), and rabbit-anti-TGN46 (1:100, Sigma Aldrich, polyclonal). Cells were washed in PBS three times before adding secondary antibody. Secondary antibodies were diluted (1:400) in 5% bovine serum albumin in PBS containing 0.01% TritonX-100 and cells were stained for 2 h. Secondary antibodies included Alexa Fluor 647 goat anti-mouse (Jackson ImmunoResearch) and Alexa Fluor 647 donkey anti-rabbit (Jackson ImmunoResearch). Mitochondria were stained for 30 min by adding 100-nM MitoTracker^®^ Deep Red FM (Thermo Fisher Scientific) to CD4 HeLa cells cultured in 12-well plates. Cells were then fixed as above and coverslips were mounted on slides using Fluoromount-G or 4′,6-diamidino-2-phenylindole (DAPI)-Fluoromount-G (Southern Biotech, Birmingham, AL, USA). Cells were imaged on a Leica DMI6000 B at 100× objective settings using a Hamamatsu Orcaflash 4.0 Camera. Images were deconvolved using the Leica Application Suite software. Colocalization analysis was conducted using the Pearson Correlation from the Image J JaCoP plugin [35], as previously described [18,36]. Statistical analysis comparing Pearson’s correlations amongst subtypes with the various subcellular markers was completed using GraphPad Prism with a one-way Anova with Tukey’s multiple comparisons test. Surface modeling of the Nef G (FI.93.HH8793):MitoTracker^®^ colocalization was generated via three-dimensional (3D) rendering using the Imaris software (version 7.0; Bitplane, Concord, MA, USA).

### 2.6. Quantitative RT-PCR

Jurkat E6.1 cells were infected with lentiviral vectors [33] encoding Nef NL4.3 or Nef from subtype G (FI.93.HH8793) or H (BE.93.VI997) reference strains. Forty-eight hours post infection, RNA was collected with the PureLink^®^ RNA Mini Kit (Thermo Fisher Scientific). The cDNA was reverse-transcribed from the isolated messenger RNA (mRNA) with SuperScript^®^ III First-Strand Synthesis System. The cDNA was used for qRT-PCR with a SensiFAST™ SYBR No-ROX Kit (FroggaBio) to amplify viral envelope protein (Env)-, Nef-, and glyceraldehyde 3-phosphate dehydrogenase (GAPDH)-specific amplicons with the following primers: Env and Nef forward 5′–GGCGGCGACTGAAGAAG, Env reverse 5′–ACTATGGACCACACAACTATTGCT, Nef reverse 5′–GATTGGGAGGTGGGTTGCT, GAPDH forward 5′–ACAACTTTGGTATCGTGGAAGG, and GAPDH reverse 3′–GCCATCACGCCACAGTTTC. The qRT-PCR runs were performed on a Rotor-Gene 6000 (Qiagen, Valencia, CA, USA) as previously described [33]. The relative levels of Nef, Env, and GAPDH mRNA were calculated from standard curves generated from linearized plasmids encoding the respective genes at known concentrations. Statistical analysis comparing mRNA levels was completed with GraphPad Prism using a one-way Anova with Dunnett’s multiple comparisons test.

## 3. Results

### 3.1. Nef from Reference Strains of HIV-1 Group-M Subtypes Exhibit Differential Functionality, Expression, and Subcellular Distribution

**Figure 1:**
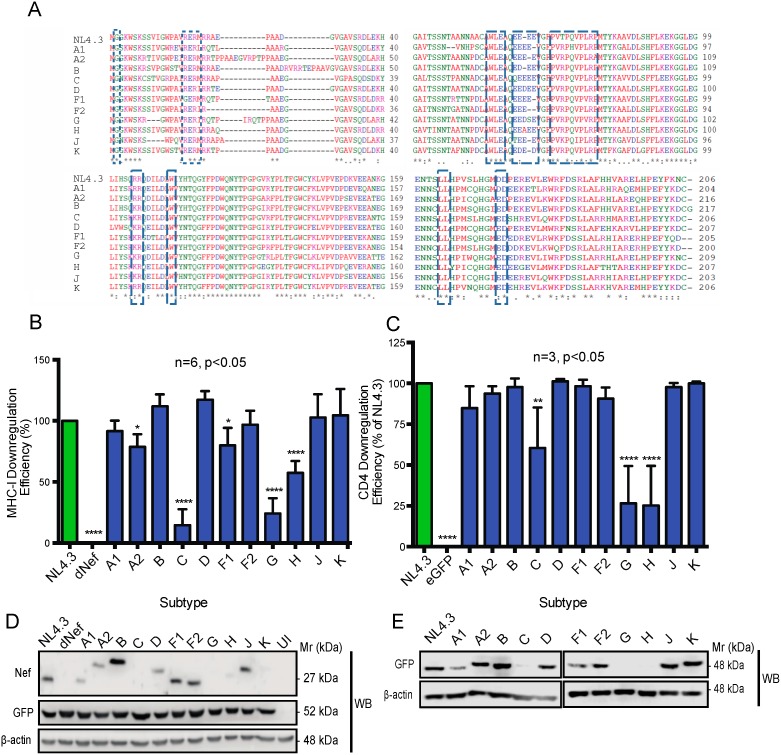
Nef from various human immunodeficiency virus type 1 (HIV-1) group-M subtype reference strains display differential expression and functionality. (**A**) Clustal Omega [39,40] was used to align Nef protein sequences from reference strains of group-M HIV-1 subtypes. * indicates identical residues, : indicates conserved residues, . indicates semiconserved residues. Residues in blue are acidic amino acids, residues in pink are basic amino acids, residues in red are small hydrophobic amino acids, and residues in green are hydroxyl, amine, or basic amino acids. Nef domains are boxed. (**B**) Isogenic NL4.3 dGag/Pol enhanced GFP (eGFP) pseudoviruses only differing in the *nef* gene from reference strains of group-M subtypes were used to infect Jurkat E6.1 T cells. Cell-surface major histocompatibility complex class I (MHC-I) levels were measured via flow cytometry 48 h post infection with a pan-specific anti-MHC-I primary antibody and an Alexa Fluor 647-conjugated secondary antibody. Downregulation efficiency is relative to NL4.3. (**C**) Plasmids encoding eGFP-tagged Nef proteins from reference strains of group-M subtypes were transfected into cluster of differentiation 4 (CD4) HeLa cells. Forty-eight hours post transfection, cell-surface CD4 levels were quantified via flow cytometry using an allophycocyanin (APC)-conjugated anti-CD4 antibody and measured using flow cytometry. Downregulation efficiency is relative to NL4.3. (**D**) Jurkat E6.1 T cells were infected with isogenic pNL4.3 dGag/Pol eGFP pseudoviruses only differing in the *nef* gene. Forty-eight hours post infection, cells were lysed and immunoblots were conducted to detect Nef expression. (**E**) Plasmids encoding Nef–eGFP fusion proteins were transfected into CD4 HeLa cells. Forty-eight hours post transfection cells were lysed, and immunoblots were conducted. A mouse anti-actin primary antibody was used to validate equal loading of protein. (UI: uninfected; dNef: pseudovirus deficient in *nef*; Mr: molecular weight; * *p* ≤ 0.05; ** *p* ≤ 0.01; **** *p* ≤ 0.0001).

The ability of HIV-1 Nef to downregulate MHC-I and CD4 from the cell surface is a hallmark of Nef function [37]. Hence, we compared the ability of Nef isolates from HIV-1 group-M subtype reference strains to downregulate MHC-I and CD4 from the cell surface to investigate the importance of genetic diversity on Nef function. Nef protein sequence alignments from various patient isolates demonstrated that Nef functional motifs involved in MHC-I and CD4 downregulation [17,38] are conserved across strains (Figure 1A). Hence, we would predict these variants to exhibit similar functionality in terms of receptor downregulation. To first investigate the ability of these Nef proteins to downregulate MHC-I, pNL4.3 dGag/Pol eGFP pseudoviruses encoding Nef variants from different group-M reference strains were used to infect Jurkat E6.1 T cells (Figure 1B). Alternatively, to investigate the ability of Nef isolates to downregulate CD4 from the cell surface, CD4 HeLa cells were transfected with pN1 Nef–eGFP plasmids encoding eGFP-tagged Nef proteins from the HIV-1 group-M subtype strains of interest (Figure 1C). Subsequently, cell surface levels of CD4 and MHC-I were measured using flow cytometry (Figure 1B,C). Flow cytometry analysis indicated that the Nef proteins from HIV-1 reference strains C (BR.92025), G (F1.93.HH8793), and H (BE.93.VI997) displayed a marked reduction in the ability to downregulate both MHC-I and CD4 from the cell surface, compared to the efficiency observed with Nef isolated from the laboratory strain NL4.3 (Figure 1B,C). The decreased ability of C (BR.92025) to downregulate MHC-I and CD4 is consistent with our previous report [33]. Alternatively, the Nef proteins of reference from subtypes A1, B, D, F2, J, and K display a similar ability to downregulate MHC-I when compared to NL4.3, while Nef proteins of reference from subtypes A1, A2, B, D, F1, F2, J, and K display a similar ability to downregulate CD4 when compared to NL4.3 (Figure 1B,C).

We next sought to investigate if the various Nef proteins exhibited differences in expression. Nef expression was examined via immunoblot of Jurkat E6.1 T cells infected with pNL4.3 dGag/Pol eGFP pseudoviruses expressing the various Nef proteins (Figure 1D). Immunoblotting for Nef revealed that the tested subtypes A1 (SE94), C (BR.92025), G (F1.93.HH8793), H (BE.93.VI997), and K (CD97EQTB11C) reference strain Nef proteins all display reduced levels of Nef expression compared to NL4.3 (Figure 1D). However, the differences in Nef protein levels may be a result of differential binding of the anti-Nef antibody to the various Nef proteins, as the antibody was produced against a Nef protein which may be dissimilar to the diverse Nef proteins examined herein [41]. Therefore, CD4 HeLa cells were transfected with plasmids encoding eGFP-tagged Nef proteins, and the various Nef proteins were detected via anti-GFP Western blot (Figure 1E). We observed that, while the subtype K Nef protein was not detected via anti-Nef Western blot (Figure 1D), it was detected via the anti-GFP Western blot (Figure 1E). In contrast, subtypes A1 (SE94), C (BR.92025), G (F1.93.HH8793), and H (BE.93.VI997) Nef proteins exhibited reduced expression relative to NL4.3 Nef upon Western blotting with both an anti-Nef and anti-GFP antibody (Figure 1D,E), suggesting that these differences in expression are not due to potential variations in antibody binding. Notably, while the GFP-tagged Nef C, G, and H proteins were undetectable via Western blot, they were detected via flow cytometry, suggesting these proteins are expressed, albeit at a significantly lower level than Nef B–GFP (Figure S1). Moreover, the reduced protein levels observed with these Nef G and H proteins are not due to decreased mRNA levels, when compared to a high-expressing Nef (Figure S2). Low expression of the subtype C Nef protein is consistent with our previous characterization of this variant [33]; however, the reduced Nef expression from the tested reference strains A1, G, and H was not previously reported. Interestingly, despite the reduced expression relative to NL4.3 Nef (Figure 1D,E), the subtype A1 reference strain Nef was functional for both MHC-I and CD4 downregulation (Figure 1B,C). In contrast, the reduced expression and functionality of Nef from this subtype C (BR.92025) reference strain is in line with our previous report [33]. Similar to this subtype C reference stain Nef, the observed reductions in expression of Nef from the subtype G (F1.93.HH8793) and H (BE.93.VI997) reference strains may contribute to the impaired ability of these Nef proteins to downregulate MHC-I and CD4.

**Figure 2:**
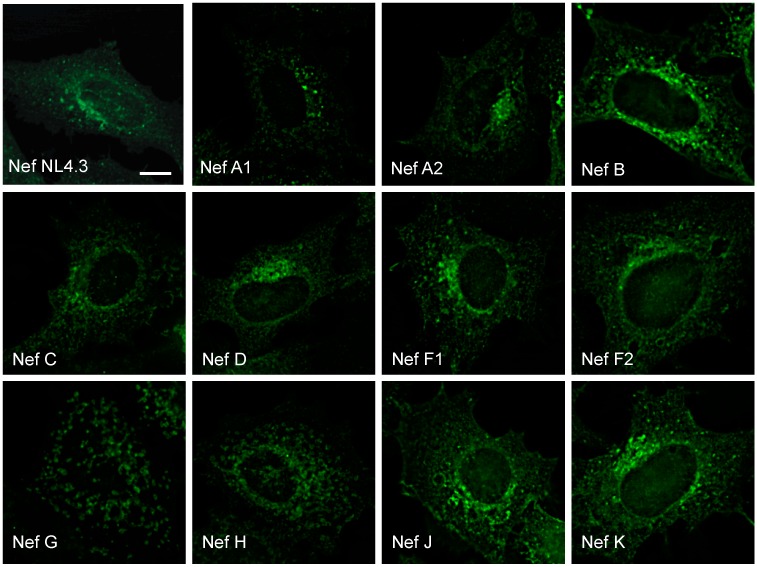
Differing subcellular distribution of Nef proteins from reference strains of HIV-1 group-M subtypes. CD4 HeLa cells were transfected with various plasmids encoding eGFP-tagged Nef proteins from reference strains of different HIV-1 subtypes (A through K). Cells were fixed in 4% paraformaldehyde and imaged on a Leica DMI6000 B widefield microscope on the 100× objective. (scale bar = 10 µm. Green = Nef–eGFP).

Subsequently, since CD4 and MHC-I receptor downregulation during HIV-1 infection are associated with Nef-dependent subcellular trafficking events, we set out to investigate the trafficking pathways utilized by Nef proteins from these various reference strains and their subcellular localization. Accordingly, HeLa cells were transfected with pN1 Nef–eGFP fusion plasmids encoding Nef proteins from reference strains of HIV-1 group-M subtypes and subjected to immunofluorescence microscopy. Reference strains of subtypes A1, A2, B, C, D, F1, F2, J, and K, and laboratory strain NL4.3 all displayed a paranuclear Nef distribution (Figure 2), consistent with previous reports of Nef localization and function [17,18,42]. However, Nef from the reference strains G (F1.93.HH8793) and H (BE.93.VI997) lacked this paranuclear distribution, and appeared more cytoplasmic, clustered and aggregated in morphology, and overall reticulated in nature (Figure 2).

**Figure 3:**
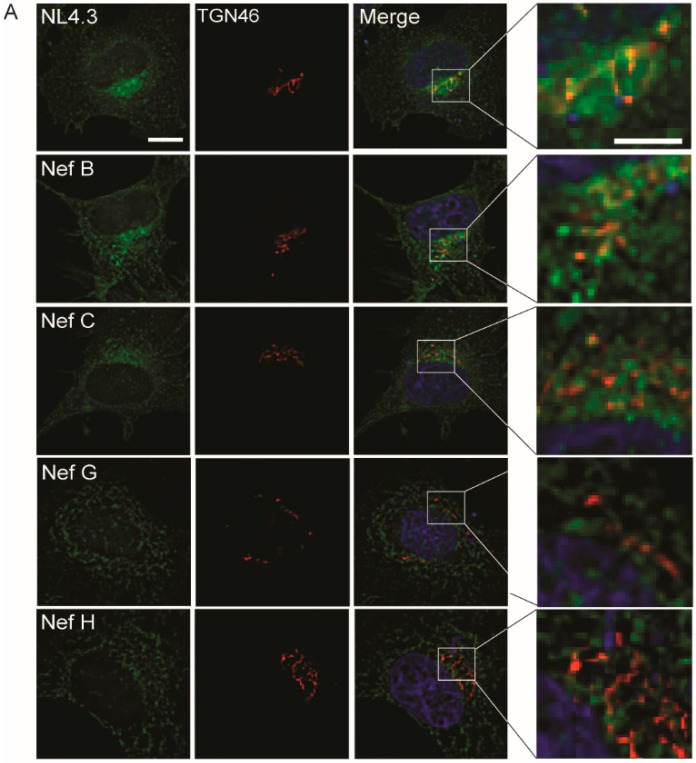

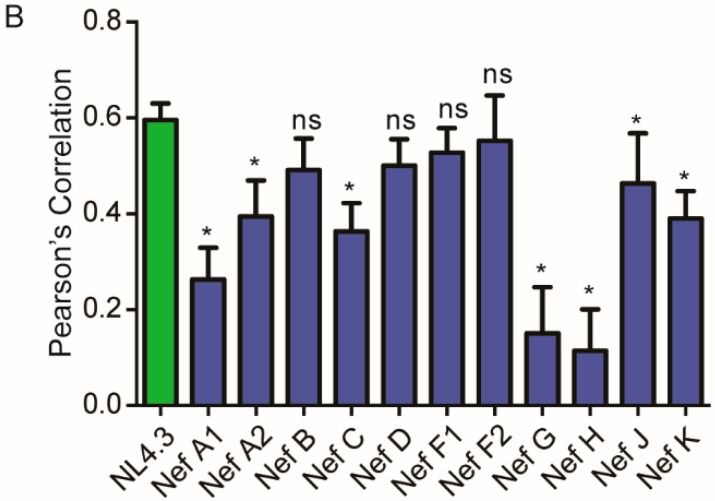
Differing trans-Golgi localization of Nef proteins from reference strains of HIV-1 group-M subtypes. (**A**) CD4 HeLa cells were transfected with plasmids encoding eGFP-tagged Nef proteins from different HIV-1 subtype reference strains. Cells were fixed in 4% paraformaldehyde, immunostained for trans-Golgi network integral membrane protein 46 (TGN46), and imaged on a Leica DMI6000 widefield microscope on the 100× objective; scale bar = 10 µm, green = Nef–eGFP, red = TGN46. Right panel insets represent a 4× magnification of the selected area. Scale bar = 5 µm. (**B**) Images were deconvolved, and colocalization analysis was completed using the Pearson’s Correlation with the JaCoP Image J plugin. Mean (+/− SD) Pearson’s correlations of Nef and TGN46 colocalization are illustrated from three independent experiments. (ns: non-significant; * *p* ≤ 0.05; SD: standard deviation).

To further characterize and quantify the differential localization of Nef proteins from the reference strains of various HIV-1 group-M subtypes, we tested if these Nef proteins localize to the TGN. Indeed, Nef traffics to the TGN, where it stimulates signaling cascades to mediate MHC-I downregulation [20]. Accordingly, HeLa cells were transfected with plasmids encoding eGFP-tagged Nef proteins from group-M reference strains, immunostained for trans-Golgi network integral membrane protein 46 (TGN46) and visualized using fluorescence microscopy. For most subtypes, Nef localized to the TGN as expected (Figure 3 and Figures S3A and S4). Specifically, Nef from laboratory strain NL4.3 and from reference strains of subtypes B, D, F1, and F2 demonstrated non-significant differences in colocalization with the TGN when compared to NL4.3 (Figure 3 and Figure S4). Although Nef from reference strains A1, A2, C, J, and K displayed less colocalization with the TGN as compared to that exhibited by NL4.3, this colocalization was still significantly greater than the colocalization observed between G (F1.93.HH8793) and H (BE.93.VI997), and the TGN (Figure 3). Indeed, colocalization analysis indicated that Nef from all subtypes except G (F1.93.HH8793) and H (BE.93.VI997) localized to the TGN with a Pearson’s correlation of approximately 0.4 or higher (Figure 3 and Figure S4). However, Nef from reference strains G (F1.93.HH8793) and H (BE.93.VI997) exhibited a drastically reduced TGN colocalization when compared to high-expressing subtypes, such as NL4.3 or Nef B (Figure 3A). Thus, Nef G (F1.93.HH8793) and H (BE.93.VI997) are localized away from the TGN, which may consequentially impact the functionality of these Nef proteins within the cell.

### 3.2. Nef Isolates from HIV-1 Reference Strains of Group-M Subtypes G and H Do Not Display Selective Colocalization with the Lysosome

**Figure 4:**
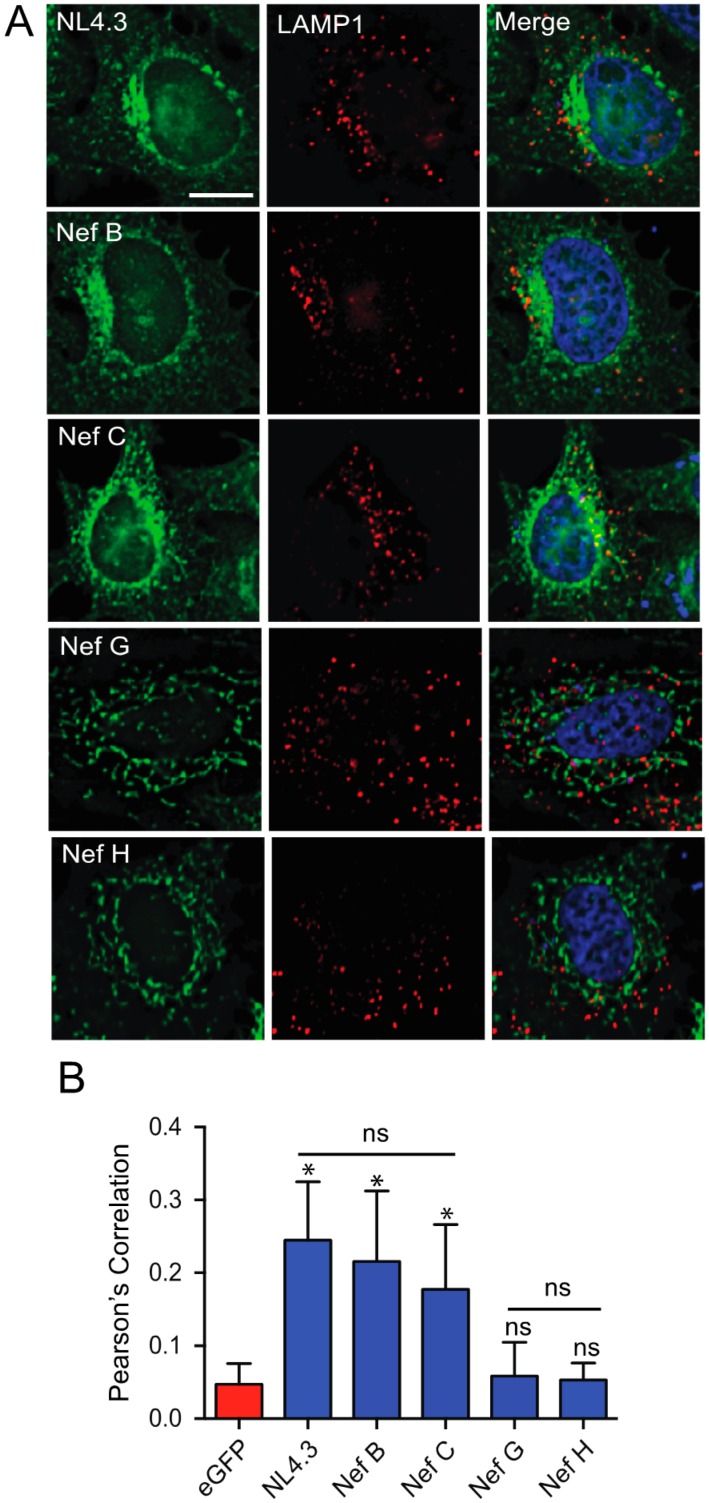
Differing lysosomal colocalization of Nef proteins from reference strains of HIV-1 group-M subtypes. (**A**) CD4 HeLa cells were transfected with various pN1 Nef–eGFP fusion plasmids encoding Nef from different HIV-1 subtypes, and immunostained for lysosome-associated membrane protein 1 (LAMP1). Cells were fixed in 4% paraformaldehyde and imaged on a Leica DMI6000 B widefield microscope on the 100× objective; scale bar = 10 µm, green = Nef–eGFP, red = LAMP1. (**B**) Images were deconvolved, and colocalization analysis was completed using the Pearson’s Correlation with the JaCoP Image J plugin. Mean (+/− SD) Pearson’s correlations of Nef and LAMP1 colocalization are illustrated from three independent experiments. Red bar = negative control. (ns: non-significant; * *p* ≤ 0.05; SD: standard deviation).

Based on our finding that Nef proteins from the G (F1.93.HH8793) and H (BE.93.VI997) strains failed to localize with the TGN (Figure 3), we sought to characterize their subcellular localization and identify where these proteins are localized. Previous reports demonstrated that Nef-mediated trafficking events can degrade cellular receptors through their selective trafficking to lysosomes [19,31,43]. Hence, we compared the ability of Nef proteins from reference strains G (F1.93.HH8793) and H (BE.93.VI997) to localize to lysosomal compartments, relative to other group-M subtypes. To test this, HeLa cells were transfected with plasmids encoding eGFP-tagged Nef from the HIV-1 laboratory strain NL4.3 or subtype B, C, G, and H reference strains, and subsequently immunostained for LAMP1 which labels lysosomes (Figure 4 and Figure S3B) [44]. Treatment with the lysosomal inhibitor, NH_4_Cl [45], ensured that Nef was not degraded prior to imaging. HIV-1 Nef NL4.3, Nef B, or Nef C individually exhibited significant colocalization with LAMP1 (Pearson’s correlation ~0.2), compared to our negative control of quantifying unfused eGFP colocalization with LAMP1 (Figure 4B). However, this level of colocalization of Nef with LAMP1 is minimal and is in agreement with expected Nef trafficking functions within the cell [18]. Furthermore, there was significantly less colocalization of HIV-1 Nef from reference strain G (F1.93.HH8793) and H (BE.93.VI997) with lysosomal compartments compared to HIV-1 Nef NL4.3, Nef B, or Nef C (Figure 4B), having Pearson’s correlations less than 0.1. Specifically, Nef from reference strains G and H colocalized with LAMP1 to no greater extent than our eGFP negative control (Figure 4B and Figure S3B), again suggesting that reference subtype G (F1.93.HH8793) and H (BE.93.VI997) Nef proteins do not traffic to lysosomes; rather, they display unique cellular localization and trafficking.

### 3.3. Nef from HIV-1 Group-M Subtypes G (F1.93.HH8793) and H (BE.93.VI997) Reference Strains Do Not Display Differential Colocalization with the Endoplasmic Reticulum 

**Figure 5:**
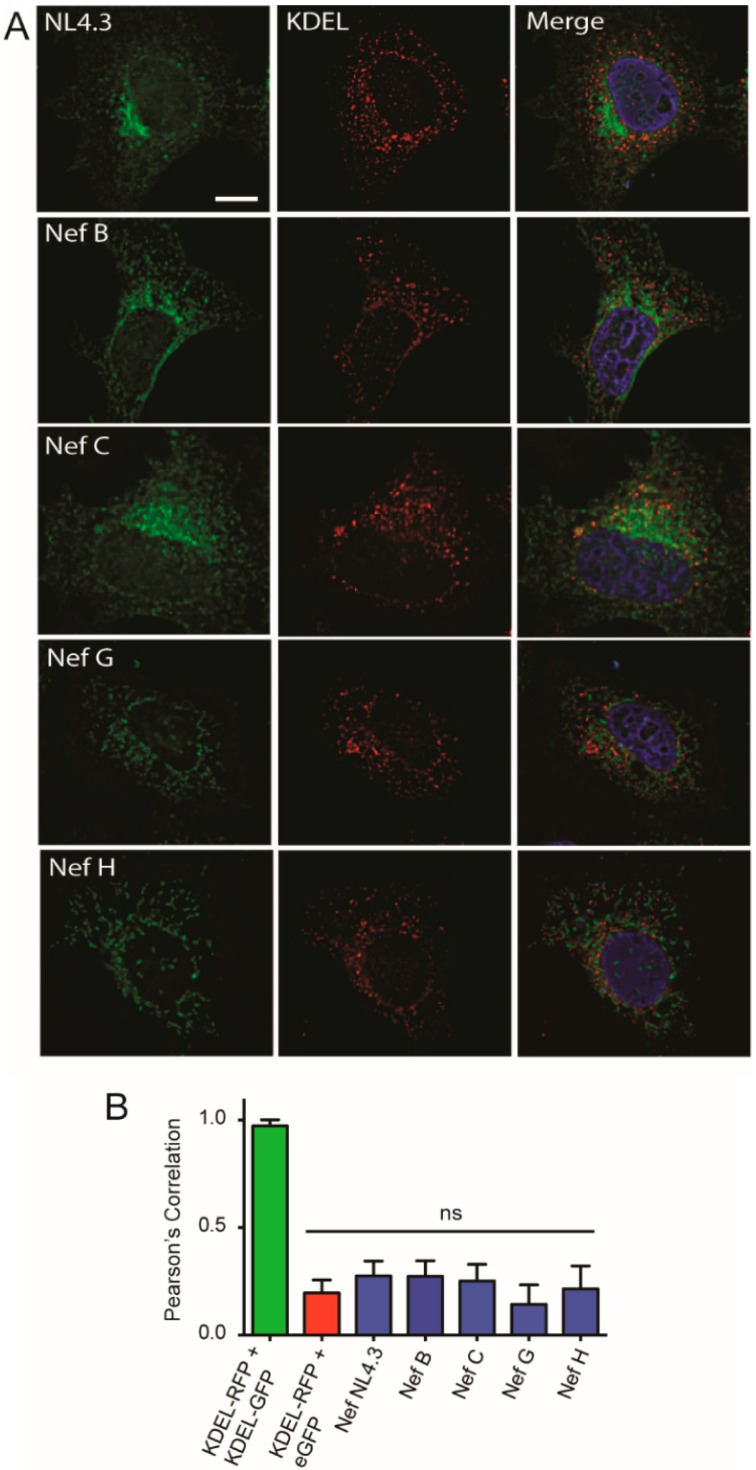
Nef proteins from reference strains of HIV-1 group-M subtypes do not localize to the endoplasmic reticulum. (**A**) CD4 HeLa cells were transfected with various Nef–eGFP fusion plasmids encoding Nef from different HIV-1 subtype reference strains or a vector encoding KDEL–red fluorescent protein (RFP). Cells were fixed in 4% paraformaldehyde, and imaged on a Leica DMI6000 widefield microscope on the 100× objective; scale bar = 10 µm, green = Nef–eGFP, red = KDEL–RFP. (**B**) Images were deconvolved, and colocalization analysis was completed using the Pearson’s Correlation with the Image J JaCoP plugin. Green bar = positive control, red bar = negative control. Mean (+/− SD) Pearson’s correlations of Nef and KDEL–RFP colocalization are illustrated from three independent experiments. (ns: non-significant; SD: standard deviation).

We next sought to determine other potential subcellular locations to which HIV-1 Nef proteins from reference strains G and H may colocalize. Hence, we investigated the possibility that these low-expressing Nef variants, G (F1.93.HH8793) and H (BE.93.VI997), colocalize with the endoplasmic reticulum (ER), as localization with the ER may occur if an unfolded protein or stress response accounted for their lower expression [46]. Indeed, based on the observed cytoplasmic, clustered, and aggregated cellular distribution of Nef G (F1.93.HH8793) and H (BE.93.VI997) (Figure 2), these proteins themselves may be cellular aggregates or stimulating ER stress responses. To examine this, a vector encoding KDEL–RFP, an ER-targeting protein [47], was cotransfected with vectors encoding eGFP-tagged Nef proteins from the laboratory strain NL4.3 and reference strains from group-M subtypes B, C, G, and H into CD4 HeLa cells. Interestingly, all tested Nef proteins displayed low levels of colocalization with the ER, comparable to our control of untagged eGFP colocalization with KDEL–RFP (Figure 5 and Figure S3C). In contrast, KDEL–RFP almost perfectly colocalized with KDEL–GFP, providing a Pearson’s correlation indicative of a positive colocalization to the ER (Figure 5B and Figure S3D). Taken together, the low-expressing HIV-1 Nef proteins from strains G (F1.93.HH8793) and H (BE.93.VI997) display minimal trafficking and colocalization with two key organelles of the secretory pathway, the TGN and ER.

### 3.4. Nef from Reference Strains of HIV-1 Group-M Subtypes G or H Do Not Colocalize with CD63

**Figure 6:**
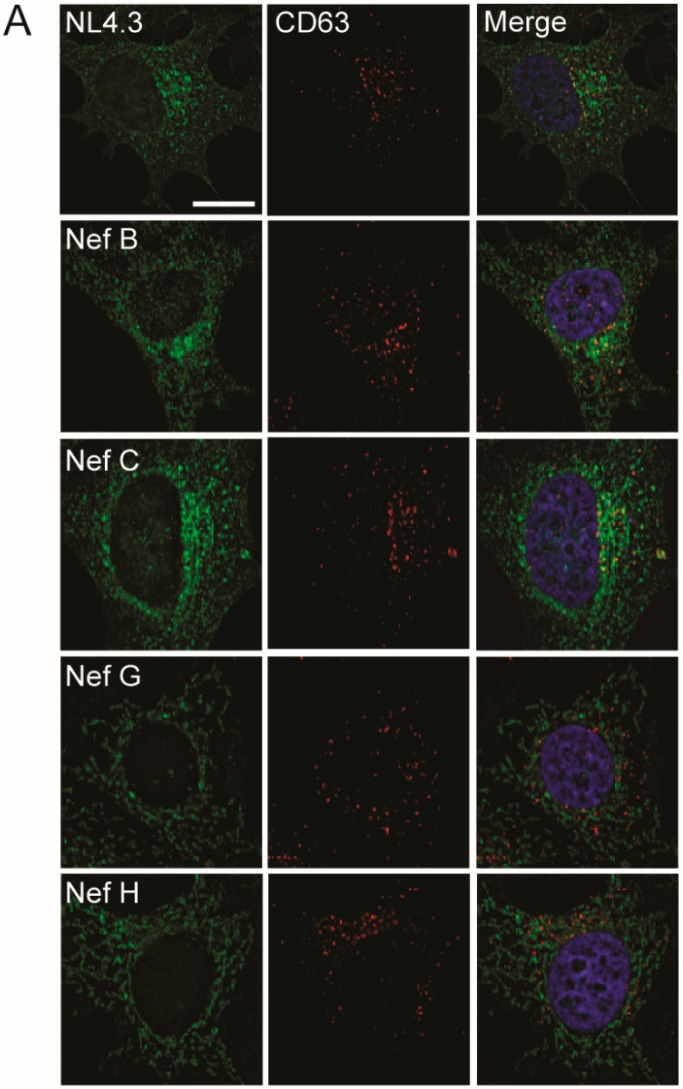

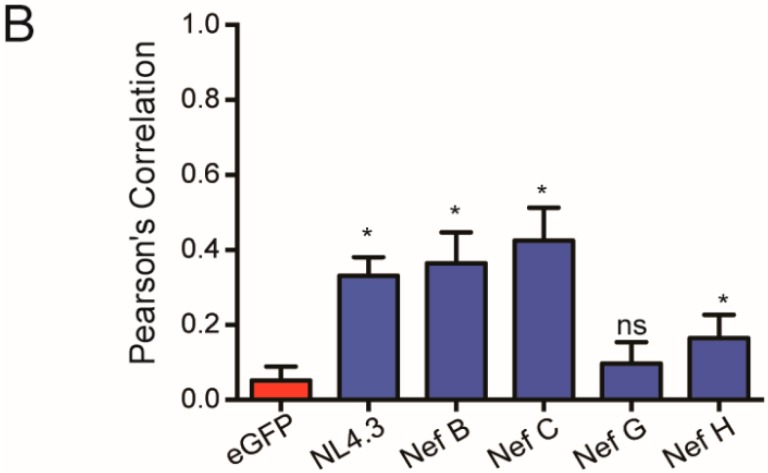
Nef from reference strains of HIV-1 group-M subtypes display differential levels of colocalization with CD63. (**A**) CD4 HeLa cells were transfected with various Nef–eGFP fusion plasmids encoding Nef from different HIV-1 subtype reference strains. Cells were fixed in 4% paraformaldehyde, immunostained for CD63, and imaged on a Leica DMI6000 widefield microscope on the 100× objective; scale bar = 10 µm, green = Nef–eGFP, red = CD63. (**B**) Images were deconvolved, and colocalization analysis was completed using the Pearson’s Correlation with the Image J JaCoP plugin. Mean (+/− SD) Pearson’s correlations of Nef and CD63 colocalization are illustrated from three independent experiments. Red bar = negative control. (ns: non-significant; * *p* ≤ 0.05; SD: standard deviation).

Having examined localization with compartments within the common routes of protein trafficking, degradation, and sequestration, we next investigated Nef localization with compartments involved in vesicular trafficking and protein secretion. It was previously reported that Nef can be secreted from cells in exosomes [48,49,50,51]. Therefore, we investigated the possibility that Nef proteins from reference strains G (F1.93.HH8793) or H (BE.93.VI997) may uniquely localize to compartments implicated in exosome formation and trafficking [49]. Accordingly, HeLa cells were transfected with vectors encoding Nef proteins from different group-M subtype strains, and were immunostained for CD63. CD63 is a tetraspanin protein that labels multivesicular bodies, a compartment of the endocytic network which produce vesicles destined for cellular degradation, or for secretion in the form of exosomes (Figure 6 and Figure S3E) [52].

Interestingly, Nef from laboratory strain NL4.3, and reference strains B and C (BR. 92025) colocalized with CD63, displaying Pearson’s correlations of ~0.4 (Figure 6B). Nef C colocalized with CD63 slightly more than high-expressing Nef B (Figure 6B). Notably, reference strain Nef G and H display minimal colocalization with CD63 compared to Nef NL4.3, Nef B, or Nef C, again suggesting Nef G and H have different cellular trafficking and localization. In either case, it does not appear that there is localization of Nef from HIV-1 isolates G (F1.93.HH8793) or H (BE.93.VI997) to multivesicular bodies or the canonical host vesicular trafficking system.

### 3.5. Nef from Reference Strains of HIV-1 Group-M Subtypes G (F1.93.HH8793) and H (BE.93.VI997) Selectively Colocalize with Mitochondria

Having explored multiple cellular pathways through which Nef proteins could exert trafficking modulation, it was observed that the subcellular reticulated distribution of Nef from strains G (F1.93.HH8793) and H (BE.93.VI997) may closely resemble the subcellular distribution of mitochondria.

**Figure 7:**
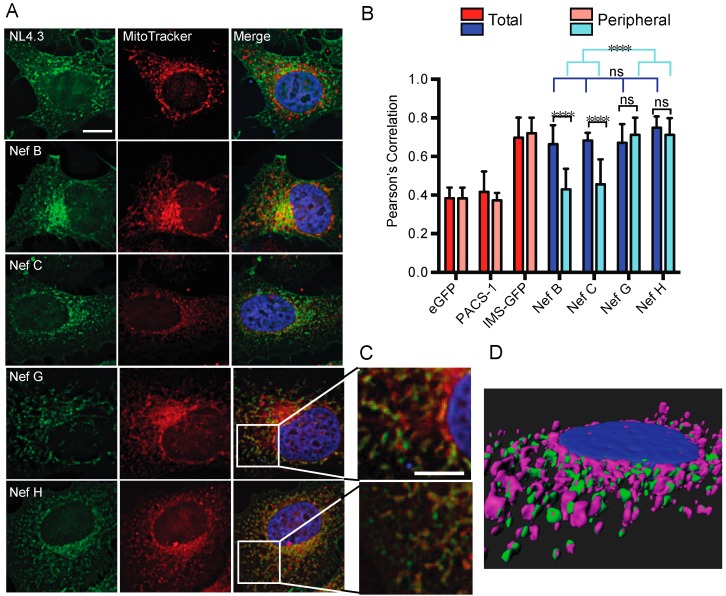
Nef from HIV-1 group-M reference strains G (F1.93.HH8793) and H (BE.93.VI997) colocalize with MitoTracker^®^. (**A**) CD4 HeLa cells were transfected with various pN1 Nef–eGFP fusion plasmids encoding Nef from different HIV-1 subtype reference strains. Twenty-four hours post transfection, cells were stained with 100-nM MitoTracker^®^ DeepRed for 15 min, then fixed in 4% paraformaldehyde, and imaged on a Leica DMI6000 widefield microscope on the 100× objective; scale bar = 10 µm, green = Nef–eGFP, red = MitoTracker^®^. (**B**) Total and peripheral cellular colocalization of Nef–eGFP and MitoTracker^®^ are shown. Images were deconvolved, and colocalization analysis was completed using the Pearson’s Correlation with the JaCoP Image J plugin. Mean (+/− SD) Pearson’s correlations of Nef and MitoTracker^®^ DeepRed colocalization are illustrated from three independent experiments. Red bars = controls; blue bars = experimental samples. (**C**) Right panel insets represent a 4× magnified image of the area selected. Scale bar = 5 µm. (**D**) A sample surface modeling of the Nef G–MitoTracker^®^ colocalization was generated via a three-dimensional (3D) rendering using the Imaris softeare (version 7.0); green = Nef, pink = mitochondria (ns: non-significant; **** *p* ≤ 0.0001; SD: standard deviation).

To test this, we transfected HeLa cells with plasmids encoding Nef–eGFP fusion proteins from the laboratory strain NL4.3, or reference strains from group-M subtypes B, C, G, or H. Twenty-four hours post transfection, cells were stained with 100-nM MitoTracker^®^ dye, which selectively labels mitochondria [53]. Unexpectedly, Nef from all tested subtypes colocalized with MitoTracker^®^ when calculating the total cellular colocalization (Pearson’s correlation ~0.7; Figure 7A,B).

This colocalization of Nef with MitoTracker^®^ was significantly greater for all reference Nef proteins than the colocalization observed with our negative controls, eGFP alone or PACS-1–eGFP, a protein that does not localize to mitochondria (Figure 7B and Figure S5A,B) [54,55]. However, it is important to note that even cells transfected with unfused eGFP displayed moderate colocalization with MitoTracker^®^ (Figure 7B). We believe that this is due to the diffuse nature of the MitoTracker^®^ dye, which was particularly evident in the perinuclear region of cells (Figure 7A). Importantly, the total level of colocalization observed with all eGFP-tagged Nef proteins was similar to that seen with the positive control, GFP-tagged intermembrane space protein (IMS–GFP), which contains a mitochondrial import sequence (Figure 7B and Figure S5C) [34].

To determine if the strong colocalization of Nef with MitoTracker^®^ was maintained outside these heavily labeled regions, we selected peripheral areas of transfected cells and reanalyzed them for Nef–MitoTracker^®^ colocalization. This peripheral colocalization analysis examined protein distribution outside of the perinuclear region. Accordingly, we observed significant differences in Nef protein colocalization with MitoTracker^®^ across reference strains (Figure 7B), with Nef from reference strains G (F1.93.HH8793) and H (BE.93.VI997) retaining a Pearson’s correlation of ~0.7 (Figure 7A,B). This peripheral mitochondrial localization was also observed with the mitochondrial targeted IMS protein, but was absent with the PACS-1–GFP or eGFP negative controls (Figure 7B and Figure S4). Morphologically, labeled mitochondria in Nef G- (F1.93.HH8793) and H- (BE.93.VI997) expressing cells appeared much more spindle-like in appearance than in cells transfected with high-expressing HIV-1 Nef subtypes (Figure 7C). Moreover, Nef from reference strains B and C exhibited marked reductions in mitochondrial colocalization outside of the paranuclear region, having Pearson’s correlations of ~0.4, which is not significantly different from eGFP–MitoTracker^®^ colocalization (Figure 7A,B). Additionally, Nef from reference subtypes G and H displayed a significantly greater colocalization with labeled mitochondrial compartments, compared to B and C, outside of the paranuclear region. Of note, Nef G (F1.93.HH8793) and H (BE.93.VI997) also colocalized with MitoTracker^®^ located outside the paranuclear region (Pearson’s correlation ~0.6) in transduced HeLa cells, whereas this degree of colocalization was lost in the periphery of cells transduced with viruses expressing Nef C or Nef B proteins (Figure S6). The latter demonstrates that identical subcellular localization was observed upon Nef overexpression and infection. Interestingly, similar to the 24 h post-transfection time point (Figure 7), at 12 h post transfection, Nef G (F1.93.HH8793) and H (BE.93.VI997) also exhibited significantly higher colocalization with MitoTracker^®^ in the periphery, relative to eGFP, unlike NL4.3 Nef, Nef B (B.JRFL), or Nef C (BR. 92025). Indeed, a Pearson’s correlation of ~0.6–0.7 was observed for Nef G (F1.93.HH8793) and H (BE.93.VI997), suggesting that the trafficking of Nef G (F1.93.HH8793) and H (BE.93.VI997) to mitochondria was equivalent at the time points tested (Figure S7). Overall, a representative surface model of the Nef–MitoTracker^®^ interaction from reference strain G (F1.93.HH8793) demonstrated this high degree of colocalization (Figure 7D).

We next sought to verify if the pattern of subcellular distribution observed with the Nef G (F1.93.HH8793) and H (BE.93.VI997) proteins is also observed with Nef proteins derived from other subtype G and H viruses. To do this, we examined HeLa cells via microscopy 24 h post transfection with expression vectors encoding eGFP-tagged Nef proteins from the G (BE.1996.D) and H (CF.1990.05) viral isolates (Figure S8). Unlike with the Nef G (F1.93.HH8793) and H (BE.93.VI997) proteins, the Nef G (BE.1996.D) and H (CF.1990.05) proteins did not have a reticulated appearance (Figure S8A). Moreover, staining with TGN46 indicated that the Nef G (BE.1996.D) and H (CF.1990.05) proteins localize with this TGN marker, similar to NL4.3 Nef. Indeed, the mean Pearson’s correlations quantifying the colocalization between the Nef G (BE.1996.D) and H (CF.1990.05) proteins and TGN46 were ~0.4, indicating that these Nef proteins colocalize with the TGN. Notably, while the colocalization between NL4.3 Nef and TGN46 was significantly higher than that observed with Nef G (BE.1996.D) and H (CF.1990.05) (Figure S8B), this difference is smaller than that seen with the colocalization between TGN46 and NL4.3 and Nef G (F1.93.HH8793) and H (BE.93.VI997) (Figure 2). Given this apparent difference in the subcellular distribution between these Nef proteins derived from viruses of the same subtype, we next examined if these proteins exhibit amino-acid sequence differences (Figure S8C,D). Indeed, there are differences in the Nef protein sequences from viruses of the same subtype, which could account for differences in subcellular distribution. Therefore, at least compared to two other subtype G and H Nef proteins, the Nef G (F1.93.HH8793) and H (BE.93.VI997) proteins are unique in their reticulated appearance and lack of a distinct localization to the TGN. Overall, this phenomenon magnifies the functional diversity that is observed between different HIV-1 Nef protein sequences.

## 4. Discussion

Our results highlight the unique cellular trafficking and localization of Nef proteins from HIV-1 group-M subtype reference strain G (F1.93.HH8793) and strain H (BE.93.VI997) throughout the membrane-trafficking machinery. The reduced expression and differential cellular localization of select Nef proteins from reference strains of HIV-1 group-M subtypes (Figure 1 and Figure 2), and the reduced ability of these Nef proteins to downregulate CD4 and MHC-I receptors from the cell surface (Figure 1B,C) highlight the importance of further elucidating the impact of viral genetic diversity on key players of viral pathogenesis at the cellular level. Indeed, such differences in expression and functionality of Nef isolates from reference strains of HIV-1 group-M subtypes were unexpected considering the similarities in sequence homology and conservation of functional motifs amongst reference strains (Figure 1A).

In this report, we observed the reduced expression and function of three HIV-1 Nef proteins, Nef C (BR.92025), G (F1.93.HH8793), and H (BE.93.VI997) (Figure 1), a phenomenon that we previously observed in Nef C (BR.92025) due to this protein’s high turnover [33]. Herein, we also illustrate that this Nef C protein exhibits a subcellular localization similar to NL4.3 Nef, while the low-expressing and non-functional G (F1.93.HH8793) and H (BE.93.VI997) Nef proteins are unique in that they exhibit a differential subcellular localization. Thus, in the case of the Nef C (BR.92025) protein, the reduced functionality is not due to an alteration in the subcellular localization, in contrast to the G (F1.93.HH8793) and H (BE.93.VI997) Nef proteins. The decreased paranuclear subcellular distribution (Figure 2) and reduced colocalization with the trans-Golgi network displayed by Nef variants G (F1.93.HH8793) and H (BE.93.VI997) (Figure 3) is in stark contrast with the reported subcellular localization of HIV-1 Nef [19,38]. As previously described, an important cellular function of Nef is canonically considered to involve hijacking the host cellular membrane-trafficking machinery to traffic to the TGN via interactions with membrane-trafficking proteins, such as PACS-2 [20,23]. Indeed, the Nef–PACS-2 interaction activates Src family kinases and zeta-chain-associated protein kinase 70 (ZAP-70), which, in turn, induces phosphoinositide 3-kinase (PI3K)-dependent signaling cascades resulting in downregulation of MHC-I from the cell surface [21]. However, patient strains G (F1.93.HH8793) and H (BE.93.VI997) divert from this trafficking pattern.

The striking finding that Nef proteins from G (F1.93.HH8793) and H (BE.93.VI997) strongly colocalize with labeled mitochondrial compartments throughout the cell (Figure 7) suggests that Nef may engage in distinct functions to facilitate HIV-1 viral pathogenesis unique from Nef-mediated receptor downregulation. The possibility that these variants have an impaired ability to function in pathogenesis also exists. Moreover, the observed mitochondrial localization and reduced levels of expression of Nef G (F1.93.HH8793) and H (BE.93.VI997) (Figure 1D,E, Figure 2 and Figure 7), may potentially point to a mitochondrial-dependent initiation of autophagy, otherwise known as mitophagy. Mitophagy is an example of an organelle-specific initiation of autophagy. A variety of organelles, including the nucleus, mitochondria, plasma membrane, and endoplasmic reticulum, provide autophagic substrates or are self-degraded via autophagy to reverse homeostatic perturbations, such as viral infections [56]. Perturbations sensed at the mitochondria stimulate it to trigger mitophagy during a stress response [57]. During viral infection, autophagy is often initiated by the binding of viral nucleic acids or proteins to certain pattern-recognition receptors which are located at the interface of the mitochondria and endoplasmic reticulum [56,58,59].

Intriguingly, other viral proteins were reported to associate with mitochondria-associated membranes (MAMs) which form at the interface between the mitochondria and endoplasmic reticulum [60]. Specifically, viral proteins from both human cytomegalovirus and hepatitis C virus are reported to traffic to mitochondria [61,62]. The trafficking of viral proteins to MAMs is believed to enable viral manipulation of integral host cellular pathways such as calcium signaling, lipid synthesis, bioenergetics, and metabolism, as well as apoptosis and immune evasion, to facilitate pathogenesis and infection [61,63]. Indeed, the HIV-1 accessory protein Vpr was reported to localize to MAMs and alter their morphology [60,64]. Hence, it is not unreasonable to predict that the accessory protein, Nef, may participate in a similar role.

Moreover, in our study, when peripheral mitochondrial regions of cells were considered, the degree of colocalization of Nef proteins from subtype B and C reference strains with MitoTracker^®^ decreased to levels not significantly different from cells transfected with eGFP alone (Figure 7). Yet, the strong colocalization of Nef proteins from subtype G and H with MitoTracker^®^ was maintained. These findings support our early observations that Nef proteins from subtype G and H reference strains display unique subcellular distribution and trafficking, even when compared to the other low-expressing subtype C reference strain. They also raise the possibility that these Nef proteins are preferentially localized to mitochondria-dense regions of cells, an as of yet unreported finding for HIV-1 Nef. Indeed, this is the first report of HIV-1 Nef trafficking to suspected mitochondrial compartments.

These results highlight the importance in understanding how HIV-1 viral diversity may impact in vitro and in vivo experiments investigating HIV-1 pathogenesis, and the need to investigate strains from multiple HIV-1 group-M subtypes to gain an accurate understanding of the molecular behaviors of Nef from differing subtypes. In fact, the Los Alamos HIV-1 Database (http://www.hiv.lanl.gov/) describes strains G (F1.93.HH8793) and H (BE.93.VI997) as being representative of HIV-1 subtype G and H, respectively. Considering Nef proteins from reference strains G (F1.93.HH8793) and H (BE.93.VI997) localized to mitochondria (Figure 7), whereas Nef proteins from strains G (BE.1996.D) and H (CF.1990.05) predominantly localized to the TGN (Figure S8), this suggests that there are differences in Nef localization between strains even within a single group-M subtype. These differences are most likely due to the amino-acid variations present within strains (Figure S8). This work suggests that not all HIV-1 reference strains may be representative of the cellular behavior within an HIV-1 group-M subtype as a whole, indicating that multiple strains from each subtype should be tested to establish a reference strain. Overall, this emphasizes how much is yet to be understood about the impact of HIV-1 genetic diversity and complexity on the infectious cycle and viral protein function, and highlights the critical importance of examining multiple HIV-1 group-M subtypes. However, little information is known about subtypes G and H. Indeed, the Los Alamos HIV-1 database provides over 3000 reference strains for subtype B; however, only 90 subtype G and nine subtype H reference strains are characterized. As subtype G accounts for ~5% of global infection [2], which represent millions of infected individuals, this highlights the need for further characterization of less prevalent HIV-1 subtypes.

In conclusion, this study builds the foundation for future investigations seeking to elucidate the cellular pathways unique to Nef variants from various HIV-1 subtypes. Moving forward, understanding the variabilities in cellular behavior exhibited by HIV-1 proteins across group-M HIV-1 subtypes during viral infection will be critical for advancing the field to a more personalized understanding—and personalized treatment regimen—of HIV-1 infection.

## Supplementary Materials

The following are available online at http://www.mdpi.com/1999-4915/10/9/493/s1. Figure S1: Expression of eGFP-tagged Nef proteins in Jurkat E6.1 cells; Figure S2: Nef mRNA levels in transduced T cells; Figure S3: Representative images of experimental controls; Figure S4: Representative images of cells expressing GFP-tagged Nef reference strain proteins and stained for TGN46; Figure S5: Representative images of cells transfected with control proteins and stained with MitoTracker; Figure S6: Nef-eGFP from HIV-1 Group M reference strains G (F1.93.HH8793) and H (BE.93.VI997) colocalize with MitoTracker® in transduced cells; Figure S7: Nef from HIV-1 Group M reference strains G (F1.93.HH8793) and H (BE.93.VI997) colocalize with MitoTracker® twelve hours post-transfection; Figure S8: Subcellular localization and sequence of distinct subtype G (BE.1996.D) and H (CF.1990.05) Nef proteins.
