# Supplementary material for: Identification of Novel Subcellular Localization and Trafficking of HIV-1 Nef Variants from Reference Strains G (F1.93.HH8793) and H (BE.93.VI997)
Source: Viruses. 2018 Sep 13;10(9):493. doi: 10.3390/v10090493 (PMC6164931; doi:10.3390/v10090493)
Supplement: Supplementary file 1 [file viruses-10-00493-s001.pdf]

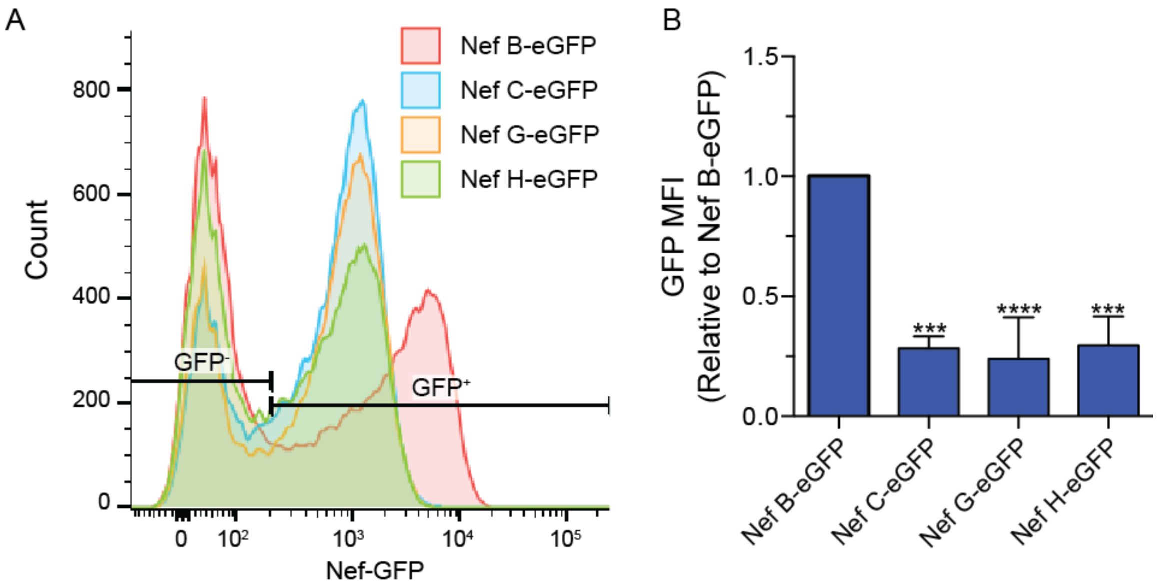

Figure S1: Expression of eGFP-tagged Nef proteins in Jurkat E6.1 cells. a) Jurkat E6.1 cells were infected with lentiviral vectors encoding Nef fused to eGFP from reference strains from subtypes B, C, G and H. Infected cells were analyzed for GFP mean fluorescence intensity by flow cytometry. A representative histogram from three independent experiments is shown. Illustrated is the gate utilized to differentiate between GFP negative (GFP^-) and GFP positive (GFP^+) cells. [Red = Nef B(JRFL)-eGFP; Blue = Nef C(BR.92025)-eGFP; Orange = Nef G(F1.93.HH8793)-eGFP; Green = Nef H(BE.93.VI997)-eGFP]. b) Infected cells were gated on GFP fluorescence and Nef expression (MFI) was quantified relative to Nef B-eGFP from three independent experiments. Shown is the mean +/- SD MFI. (MFI: mean fluorescence intensity; ***$p \leq 0.001$; **** $p \leq 0.0001$; SD: standard deviation)

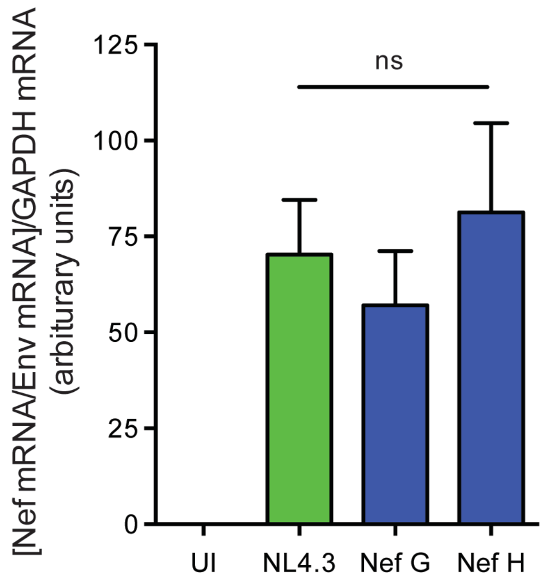

Figure S2: Nef mRNA levels in transduced T cells. Jurkat E6.1 T-cells were uninfected (UI) or infected with lentiviral vectors encoding Nef from the laboratory strain NL4.3 or Nef from the subtype reference strains G (F1.93.HH8793) or H (BE.93.VI997). At 48 hours post-infection, mRNA was isolated from the cells and used for quantitative reverse transcriptase PCR (qRT-PCR). Levels of mean (+/-SD) Nef specific mRNA are shown relative to levels of HIV-1 Env specific mRNA, normalized to GAPDH mRNA. Data is from two independent experiments. (ns: non-significant, Env: envelope, GAPDH: glyceraldehyde 3-phosphate dehydrogenase; SD: standard deviation)

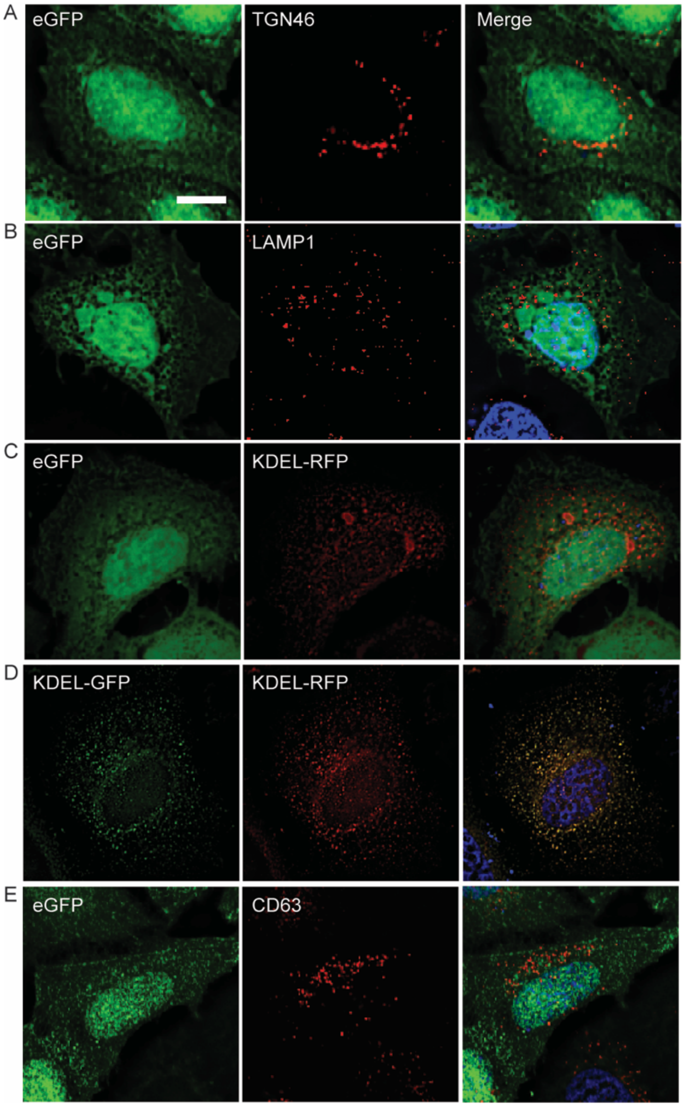

Figure S3: Representative images of experimental controls. CD4 HeLa cells were transfected with plasmids expressing eGFP, KDEL-eGFP or KDEL-RFP. Cells were fixed in 4% paraformaldehyde, immunostained for TGN46, LAMP1, CD63, or left unstained, and imaged on a Leica DMI6000 widefield microscope on the 100X objective; scale bar = 10µm, green = eGFP or KDEL-GFP (as indicated), red = indicated marker staining or KDEL-RFP.

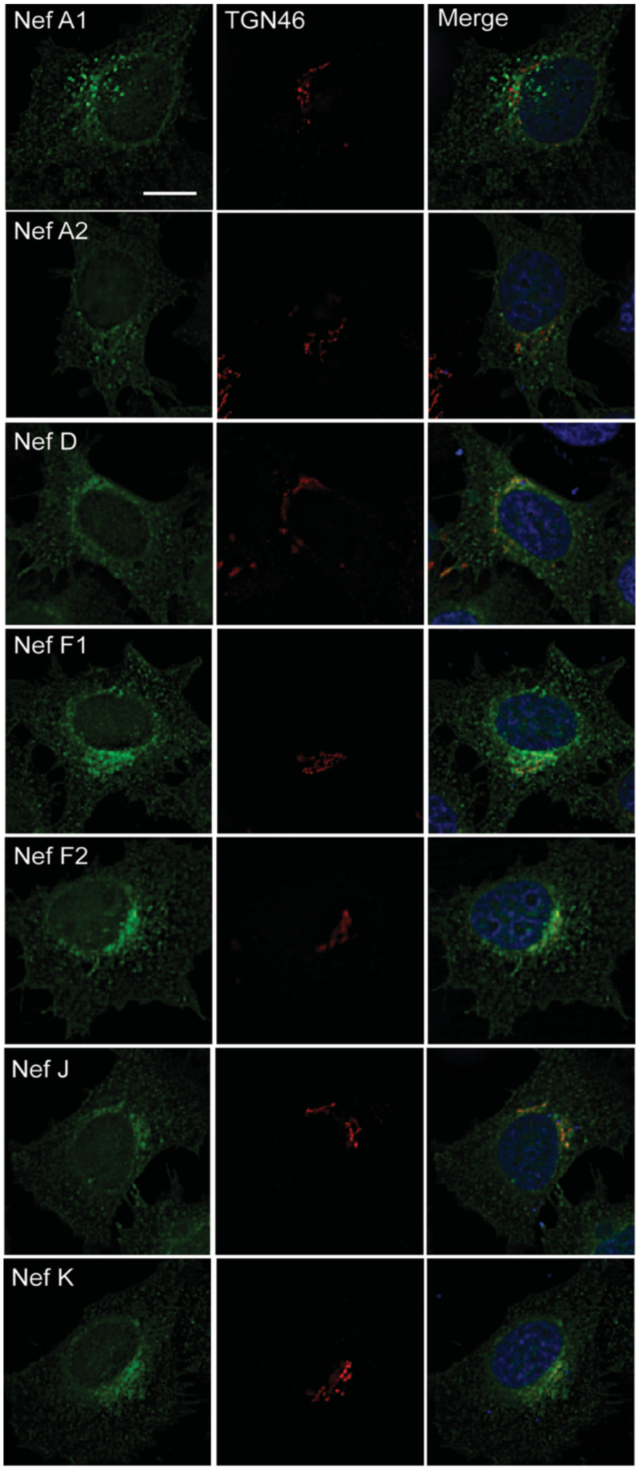

Figure S4: **Representative images of cells expressing GFP-tagged Nef reference strain proteins and stained for TGN46.** CD4 HeLa cells were transfected with plasmids encoding eGFP-tagged Nef proteins from different HIV-1 subtype reference strains. Cells were fixed in 4% paraformaldehyde, immunostained for TGN46, and imaged on a Leica DMI6000 widefield microscope on the 100X objective; scale bar = 10µm, green = Nef-eGFP, red = TGN46.

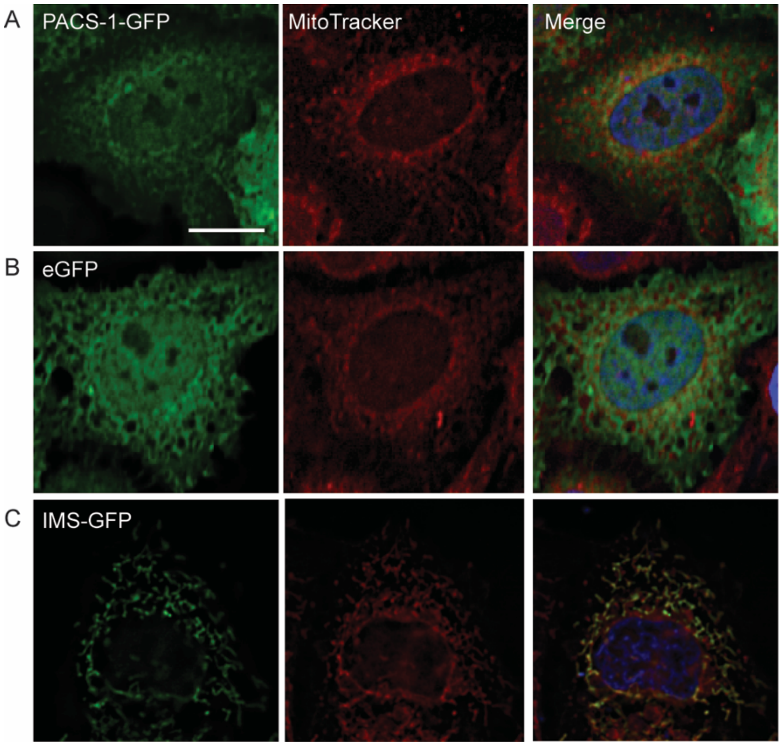

Figure S5: Representative images of cells transfected with control proteins and stained with MitoTracker. CD4 HeLa cells were transfected with plasmids encoding a) GFP-tagged PACS-1, b) eGFP or c) GFP-tagged IMS. Twenty-four hours post transfection, cells were stained with 100nM MitoTracker® DeepRed for 15min, then fixed in 4% paraformaldehyde, and imaged on a Leica DMI6000 widefield microscope on the 100X objective; scale bar = 10µm, green = eGFP, red = MitoTracker®. (PACS-1: phosphofurin acidic cluster sorting protein 1; IMS: intermembrane space protein)

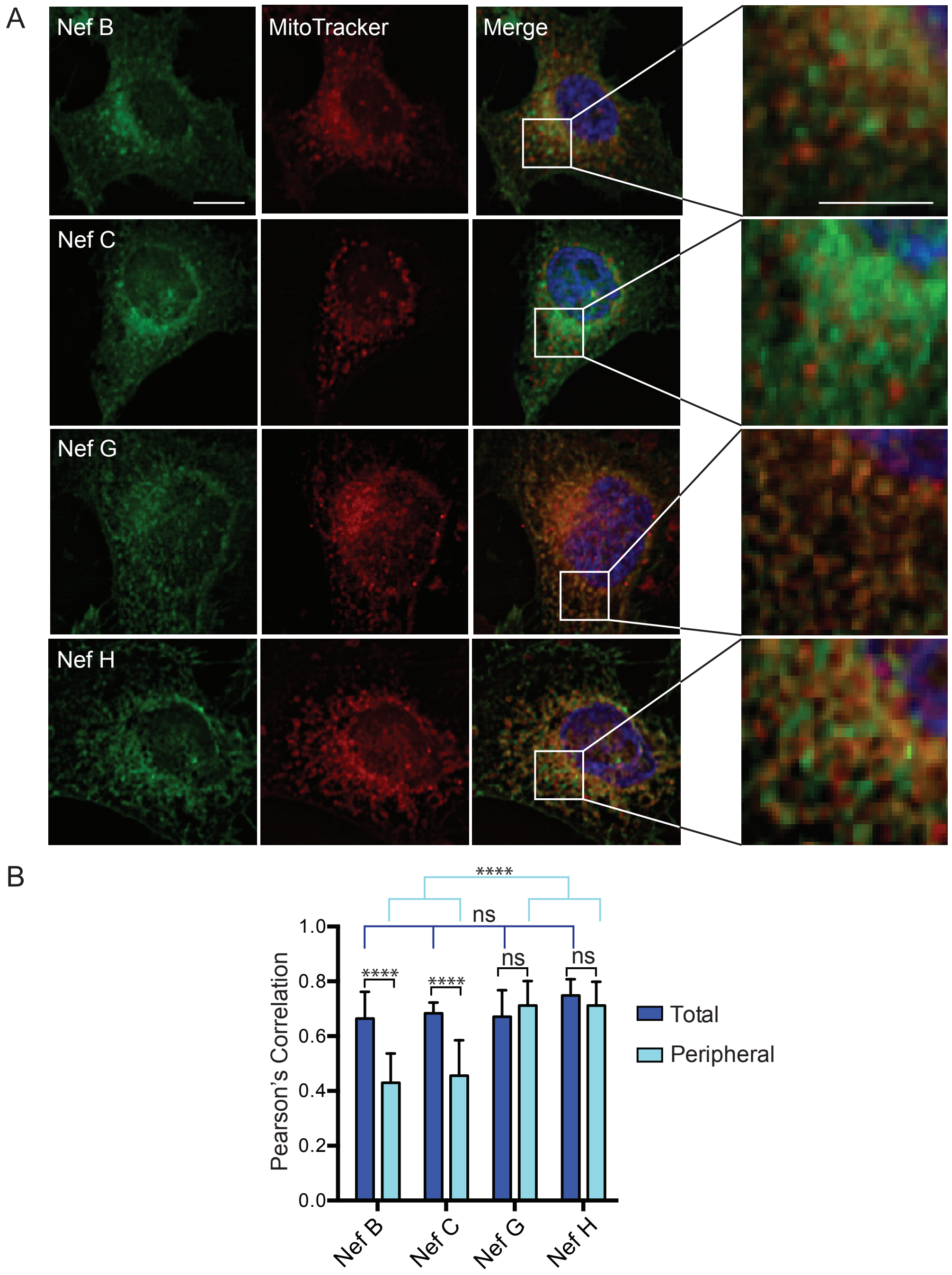

Figure S6. Nef-eGFP from HIV-1 Group M reference strains G (F1.93.HH8793) and H (BE.93.VI997) colocalize with MitoTracker® in transduced cells. a) CD4 HeLa cells were transduced with pNL4.3 dGag/Pol pseudoviruses encoding eGFP-tagged Nef variants from different group M reference strains, Nef B (B.JRFL), Nef C (C.BR92025), Nef G (F1.93.HH8793) and Nef H (BE.93.VI997). Cells were stained with 100 nM MitoTracker® DeepRed for 15min, then fixed in 4% paraformaldehyde, and imaged on a Leica DMI6000 widefield microscope on the 100X objective; scale bar = 10µm, green = Nef-eGFP, red = MitoTracker®. Right panel insets represent a 4x magnified image of the selected area. Scale bar = 5µm. b) Total and peripheral

mean Pearson's Correlation (+/-SD) of Nef-eGFP and MitoTracker® is shown from two independent experiments. Images were deconvolved and colocalization analysis was completed on either the whole cell (total; dark blue) or only within the cell periphery (peripheral; light blue) using the Pearson's Correlation with the JaCoP Image J plugin. Statistical analysis comparing means was completed with GraphPad Prism using one-way Anova with a Tukey's multiple comparisons test. Red bars = negative control. (ns: non-significant; ****$p \leq 0.0001$; SD: standard deviation)

A

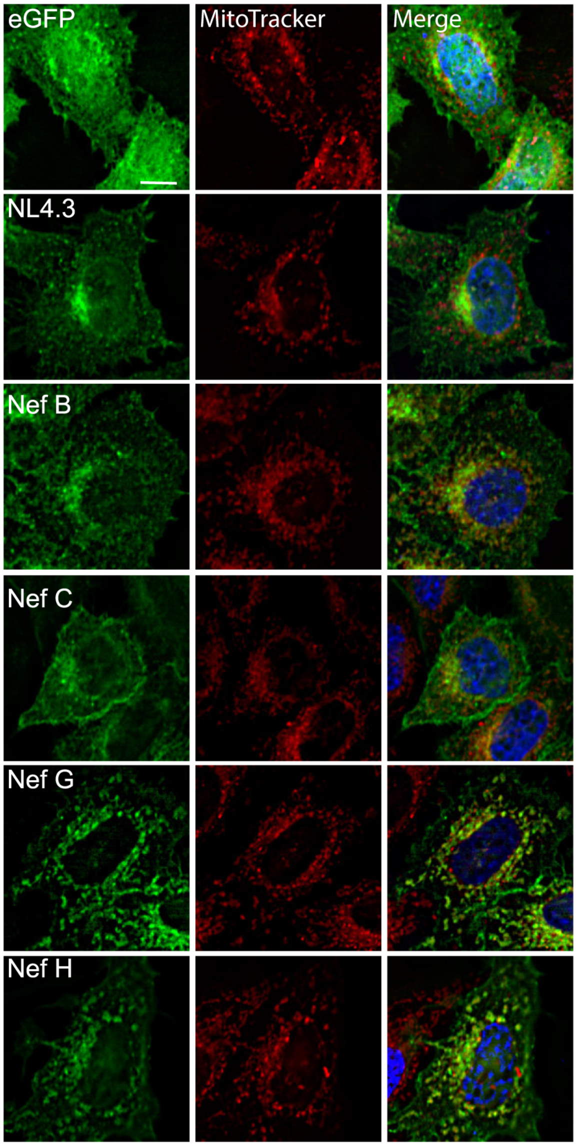
eGFP
MitoTracker
Merge
NL4.3
Nef B
Nef C
Nef G
Nef H

B

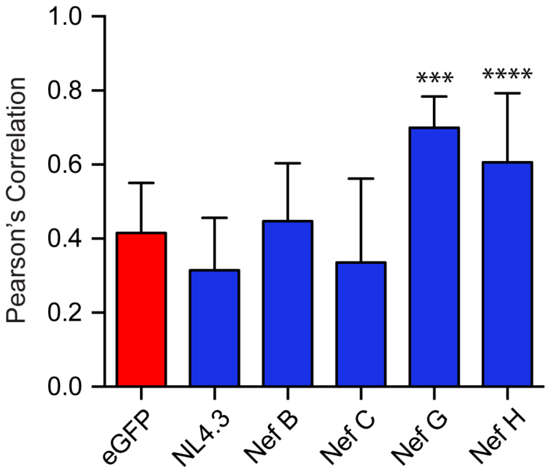
Pearson's Correlation
1.0
0.8
0.6
0.4
0.2
0.0
***
****
eGFP
NL4.3
Nef B
Nef C
Nef G
Nef H

Figure S7. Nef from HIV-1 Group M reference strains G (F1.93.HH8793) and H (BE.93.VI997) colocalize with MitoTracker® twelve hours post-transfection. a) CD4 HeLa cells were transfected with various pN1 Nef-eGFP plasmids encoding Nef from different HIV-1 subtype reference strains. Twelve-hours post-transfection cells were stained with 100nM MitoTracker® DeepRed for 15min, then fixed in 4% paraformaldehyde, and imaged on a Leica DMI6000 widefield microscope on the 100X objective; scale bar = 10µm, green = Nef-eGFP, red = MitoTracker®. b) Colocalization of Nef-eGFP and MitoTracker® in the periphery of the cell. Illustrated is the mean (+/-SD) Pearson's Correlation of Nef and MitoTracker® DeepRed colocalization from two independent experiments. Statistical analysis was completed with GraphPad Prism using one-way Anova with a Tukey's multiple comparisons test. Red bar = control; Blue bars = experimental (***$p \leq 0.001$; **** $p \leq 0.0001$; SD: standard deviation)

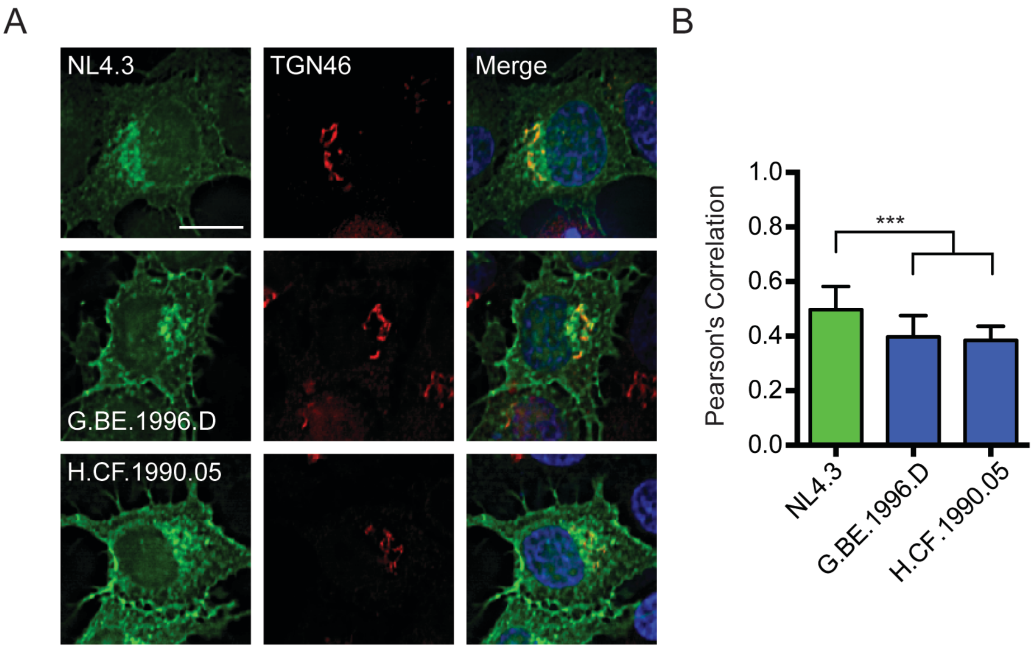

C

```
G.BE.1996.D  MGGKWSKR---GWPAVRERIRQTPIRQTPPAAEGVGAVSQDLARHGAITSSNTATNNPDC  57
G.F1.93.HH8793  MGNKWSKRKVAGWPEVRERLRQ-----HPAAAEGVGAVSQDLDRHGAITSRNTAGTNPDC  55
             **.*****   *** ****:**      * ************ ******* *** .****

G.BE.1996.D  AWLEAQEEDSEVGFPVRPQVPVRPMTYKAAFDLSFFLKEKGGLDGLIYSKKRQDILDLWV  117
G.F1.93.HH8793  AWLEAQEEDSEVGFPVRPQVPLRPMTYKAAFDLSFFLKEKGGLDGLVYSKKRQEILDLWV  115
             *********************:************************:******:******

G.BE.1996.D  YNTQGFFPDWQNYTPGPGTRLPLTFGWCLKLVPVDPAVVEEATTEENNSLLHPIWQHGME  177
G.F1.93.HH8793  YHTQGFFPDWQNYTPGPGTRVPLTFGWCFKLVPMEPSEVEEANKGENNSLLHPICQHGME  175
             *:******************:*******:****::*: ****.. ********* *****

G.BE.1996.D  DEDREVLIWRFDSSLARRHLARELHPEFYKNC  209
G.F1.93.HH8793  DEDGEVLVWRFDSSLARRHLARELHPEYYKDC  207
             *** ***:*******************:**:*
```

D

```
H.CF.1990.05  MGGKWSKSSIVGWPAVRERIRRAQPAADGVGAVSRDLDRRGAVTINNTAATNPDVAWLEA  60
H.BE.93.V1997  MGGKWSKSRMGGWSTIRERMRRAEPVAEGVGAVSRDLDRRGAVTINNTASTNRDAAWLEA  60
             ******** : ** ::***:***:*.*:*********************:** *.*****

H.CF.1990.05  QEEAEEVGFPVRPQVPLRPMTYKAALDLSHFLKEKGGLDGLIYSKKRQEILDLWVYNTQG  120
H.BE.93.V1997  QEDGEEVGFPVRPQVPLRPMTYKGAFDLSHFLKEKGGLDGLIYSKQRQDILDLWVYNTQG  120
             **:.*******************.*:*******************:**:***********

H.CF.1990.05  YFPDWQNYTPGPGEGYPLTFGWCFKLIPVDPQEVERANEGENNCLLYPICQHGMEDEEGE  180
H.BE.93.V1997  YFPDWQNYTPGPGERFPLTFGWCFKLVPVNPQEVEQANEGENNSLLHPMSLHGMEDDGRE  180
             ************** :**********:**:*****:*******.**:*:. *****:  *

H.CF.1990.05  VLMWKFDSRLAFTHTAREKHPEFYKDC  207
H.BE.93.V1997  VLMWKFDSRLALTHLARVKHPEY-KDC  206
             ***********:** ** ****: ***
```

Figure S8: Subcellular localization and sequence of distinct subtype G (BE.1996.D) and H (CF.1990.05) Nef proteins. a) CD4 HeLa cells were transfected with plasmids encoding eGFP-tagged Nef proteins from different HIV-1 subtype reference strains. Cells were fixed in 4% paraformaldehyde, immunostained for TGN46, and imaged on a Leica

DMI6000 widefield microscope on the 100X objective; scale bar = 10µm, green = Nef-eGFP, red = TGN46. b) Images were deconvolved and colocalization analysis was completed using the Pearson's Correlation with the JaCoP Image J plugin. Shown is the mean Pearson's Correlation (+/- SD) calculated from two independent experiments. Amino acid sequence alignment of (c) Nef G BE.1996.D and F1.93.HH8793, and (d) Nef H CF.1990.05 and BE.93.VI997 produced using Clustal Omega [39,40].* indicates identical residues, : indicates conserved residues, . indicates semiconserved residues. Residues in blue are acidic amino acids, residues in pink are basic amino acids, residues in red are small hydrophobic amino acids and residues in green are hydroxyl, amine or basic amino acids. (***$p \leq 0.001$; SD: standard deviation)
